# Exploitation of the vitamin A/retinoic acid axis depletes ALDH1-positive cancer stem cells and re-sensitises resistant non-small cell lung cancer cells to cisplatin

**DOI:** 10.1016/j.tranon.2021.101025

**Published:** 2021-02-05

**Authors:** Lauren MacDonagh, Rhyla Mae Santiago, Steven G. Gray, Eamon Breen, Sinead Cuffe, Stephen P. Finn, Kenneth J. O'Byrne, Martin P. Barr

**Affiliations:** a Thoracic Oncology Research Group, School of Clinical Medicine, Trinity Translational Medicine Institute, Trinity Centre for Health Sciences, St James's Hospital & Trinity College Dublin, Ireland; b Flow Cytometry Facility, Trinity Translational Medicine Institute, Trinity Centre for Health Sciences, Trinity College Dublin, Ireland; c Medical Oncology Department, St James's Hospital, Dublin, Ireland; d Histopathology Department, St James's Hospital & Trinity College Dublin, Ireland; e Cancer & Ageing Research Program, Queensland University of Technology, Brisbane, Australia

**Keywords:** Retinoic acid, Resistance, Cisplatin, Aldehyde dehydrogenase, Cancer stem cells

## Abstract

• All-*trans* retinoic acid (ATRA) and retinol significantly reduced ALDH1+ve cell subsets.
• Co-treatment of ATRA or retinol with cisplatin significantly inhibited NSCLC cell survival.
• Retinoic acid pathway inhibition induced apoptosis of chemo-resistant cells.
• RAR and RXR nuclear receptors were altered in response to ATRA.
• Targeting components of the vitamin A/retinoic acid axis can overcome cisplatin resistance in NSCLC.

## Introduction

Lung cancer is the leading cause of cancer-related deaths worldwide, where non-small-cell lung cancer (NSCLC) accounts for approximately 85% of all lung cancers. NSCLC is further divided into three histological subtypes; adenocarcinoma, squamous cell carcinoma and large cell carcinoma [1], [2], [3]. Despite the significant advances in personalised medicine and the development of therapies specifically targeting driver mutations, platinum-based chemotherapy remains the cornerstone in the current management of NSCLC since its FDA-approval in 1978 [4]. NSCLC tumours initially respond to platinum-based therapy, however despite this primary response, resistance frequently develops thereby contributing to the poor 5-year survival of <15% [5].

Cisplatin resistance is multifactorial in nature. For cisplatin to exert its cytotoxic effects, it must enter the cell and accumulate within the cell without excessive efflux, reach the DNA without being scavenged and induce cisplatin-DNA cross-links and adduct formation. A significant clinical challenge in the treatment of NSCLC patients with platinum agents, in particular cisplatin, has been the development of resistance due to a number of cellular and molecular mechanisms, numerous of which have been well described in the literature [6], [7], [8]. Alterations in cell cycle checkpoint control and apoptosis-associated protein expression have frequently been observed in cisplatin-resistant NSCLC [9,10]. More recently, the cancer stem cell (CSC) hypothesis has become a focus of platinum-induced resistance where a resilient drug-surviving subpopulation of cells endures. This surviving population in turn, has been associated with relapse following initial cytotoxic therapy [11], [12], [13]. The CSC hypothesis suggests the presence of a rare and robustly resistant cell subset within solid tumours that display key stem-like characteristics such as asymmetric division, self-renewal and differentiation, which in theory, can lead to the recapitulation of a heterogeneous tumour following treatment with chemotherapy [14,15]. CSCs have been described in numerous solid tumour types, including breast, head & neck, prostate, ovarian and lung cancer [16], [17], [18], [19], [20]. Lung cancer stem cells have previously been reported based on the expression of surface markers, such as CD133, CD44 and CD117 [18,[20], [21], [22], excessive efflux of the DNA-binding dye, Hoechst 33342 [23] and increased aldehyde dehydrogenase 1 (ALDH1) activity [19].

Studies have shown that ALDH1-positive (ALDH1+ve) cell subsets are resistant to a number of chemotherapeutic agents currently used as first-line therapy in the clinical setting and include cisplatin, gemcitabine, doxorubicin, vinorelbine and docetaxel, in contrast to ALDH1-negative (ALDH1-ve) subsets which are sensitive to the cytotoxic activity of these drugs [19]. ALDH1 overexpression is associated with poor prognosis in NSCLC patients and has been linked to a more aggressive and advanced pathological grade and stage [19]. Furthermore, increased ALDH1 expression has been associated with increased metastasis in multiple cancers, including inflammatory breast cancer [24,25].

The aldehyde dehydrogenases are part of a large family of enzymes that play a pivotal role in the metabolism of aldehydes. The human ALDH family consists of 19 isozymes that carry out important physiological and toxicological functions. The ALDH1A subfamily plays an important role in embryogenesis and development via the mediation of retinoic acid signalling [26]. The ALDH1A subfamily members include ALDH1A1, ALDH1A2 and ALDH1A3, with the ALDH1A1 isoform being most commonly associated with the tumour-initiating CSC phenotype and have been previously described and characterised in cisplatin-resistant NSCLC within our laboratory [27]. ALDH1 family members are responsible for the catalytic conversion of retinaldehyde/retinal to retinoic acid which in turn modulates cell differentiation [28]. Based on the ability of retinoic acid to induce differentiation, all-*trans* retinoic acid (ATRA) has been used as an effective therapeutic in the treatment of acute promyelocytic leukaemia (APL) [29]. ATRA induces the differentiation of immature leukaemia cells into mature, terminally differentiated granulocytes resulting in effective clinical remission in the majority of APL patients [30,31]. Recent studies have shown that inhibition of individual stemness markers such as Nanog, Oct-4 or Sox-2 in NSCLC CSCs, decreases self-renewal and tumour initiation capabilities and promotes chemotherapeutic sensitivity. This highlights the clinical potential of targeting CSCs to re-sensitise drug resistant tumour cells to chemotherapy [32], [33], [34].

Based on the current body of knowledge surrounding ALDH1 as a CSC marker, its role in the vitamin A/retinoic acid axis and its association with cisplatin resistance, we hypothesise that an effective strategy to overcome therapeutic resistance associated with CSCs could be to fully differentiate the stem-like cells into a more chemo-susceptible phenotype, similar to that observed in APL [29]. Furthermore, the role of ALDH1 in vitamin A metabolism and the production of retinoic acid could be exploited in order to drive cell differentiation via supplementation of the chemotherapeutic regimen with retinol, a substrate of the retinoic acid pathway which may further enhance the efficacy of chemotherapy in resistant tumours [35].

Previously, using an *in vitro* model of cisplatin-resistant NSCLC, we identified and characterised an ALDH1+ve CSC subset that is present across each NSCLC histological subtype [27]. While ATRA has been reported to be effective in the management of blood-based cancers, its differentiating role in relation to solid tumour CSCs has yet to be elucidated [36], [37], [38], [39]. Using an isogenic panel of cisplatin-resistant NSCLC cell lines, the *in vitro* re-sensitising capacity of retinoic acid exploitation was assessed across multiple functional parameters, including its effect on the presence of the ALDH1+ve CSC population in cisplatin-resistant lung cancer.

## Materials and methods

### Drugs

Cisplatin was dissolved in 0.15 M NaCl. Aliquots were stored at −20 °C for a maximum of 3 months and thawed immediately before use. All-*trans* retinoic acid (ATRA) was dissolved in dimethyl sulfoxide (DMSO) and stored at −20 °C. Vitamin A/retinol was dissolved in 95% (v/v) ethanol and aliquots were stored at 4 °C. ATRA and retinol were stored protected from the light. All drugs were purchased from Sigma-Aldrich.

### Cell lines

The human large cell carcinoma cell line, NCI-H460 (hereafter referred to as H460) and its resistant variant were kindly donated by Dr Dean Fennell, Centre for Cancer Research and Cell Biology, Queen's University Belfast. The human adenocarcinoma cell line, H1299, and its resistant subline were given as a gift from Dr Parviz Behnam-Motlagh, Department of Medical Biosciences, Umeå University, Sweden. The SKMES-1 squamous cell carcinoma cell line was purchased from the American Type Culture Collection (ATCC) (LGC Promochem, UK). Cisplatin-resistant (CisR) sublines were generated from each original parental (PT) cell line by continuous exposure to cisplatin, as previously described [40]. H460 and H1299 cell lines were reselected in culture with cisplatin based on IC_50_ concentrations and brought forward in the same manner as the other cell lines. Briefly, cells were treated with cisplatin (IC_50_) for 72 hr after which time cisplatin-containing media was removed and cells were allowed to recover for a further 72 hr. This development period was carried out for 6 months, after which time IC_50_ concentrations were determined and used as a maintenance dose for a further 6 months. H460 cells were grown in Roswell Park Memorial Institute (RPMI-1640) media. H1299 and SKMES-1 cells were maintained in Eagle's Minimum Essential Medium (EMEM) supplemented with 2 mM L-glutamine and 1 × non-essential amino acids (NEAA). For all cell lines, media was supplemented with 10% heat-inactivated foetal bovine serum (FBS), penicillin (100 U/ml) and streptomycin (100 μg/ml) (Lonza, UK). All cell lines were grown as monolayer cultures and maintained in a humidified atmosphere of 5% CO_2_ at 37 °C. Cell lines were routinely tested for mycoplasma infection and have previously been tested and authenticated using the PowerPlex^Ⓡ^ 16 HS System (Source BioScience, UK).

### Cell proliferation

Cell proliferation was measured using the Cell Proliferation BrdU ELISA (Roche Diagnostics Ltd., UK) according to manufacturer's instructions. Briefly, cells (H460, H1299 and SKMES-1) were seeded at 2.5 × 10^3^ cells/well in a 96-well plate. Following overnight incubation, cells were treated for 72 hr with cisplatin (0–100 μM) alone or in combination with ATRA (5 μM) or retinol (1 μM). Absorbance was recorded at 450 nm and sensitivity to cisplatin was calculated as a percentage of cell proliferation relative to untreated controls, which were set at 100%.

### Aldefluor assay

The Aldefluor assay (Stem Cell Technologies) was used to identify and isolate cell populations with ALDH1 enzymatic activity. Previous studies in our laboratory have characterised ALDH1+ve cells as CSCs, thus providing the foundation on which this CSC study has been carried out [27]. The assay was performed according to manufacturer's instructions. Briefly, cells (5 × 10^5^) were suspended in Aldefluor assay buffer containing activated Aldefluor reagent, BODIPY-aminoacetaldehyde (BAAA) for 45 min. This is a fluorescent non-toxic ALDH1 substrate that freely diffuses into intact viable cells. In the presence of ALDH1, BAAA is converted to BOPIDY-aminoacetate (BAA) which is retained within the cells expressing ALDH1. A specific ALDH1 inhibitor, DEAB, was used to inhibit the BAAA-BAA conversion and acts as an internal negative control for background fluorescence. The brightly fluorescent ALDH1+ve cells were detected using the green fluorescence channel (520–540 nm). ALDH1 activity was measured using a CyAn™ ADP flow cytometer (Dako, USA), while ALDH1+ve and ALDH1-ve fractions were sorted using a MoFlo™ XDP high speed cell sorter (Beckman Coulter, USA). For FACS analysis of ALDH1, gates were set for each sample relative to their ALDH1 inhibitor (DEAB) controls. The DEAB control is required for accurate compensation of fluorescent signals and for setting appropriate gates to discriminate between ALDH1+ve and ALDH1-ve cells in addition to background autofluorescence which varied across all parental and corresponding cisplatin resistant cell lines.

### Clonogenic survival

The survival of NSCLC cells, when challenged with cisplatin, was measured using the clonogenic survival assay. Cells were seeded at optimal cell densities and allowed to adhere overnight at 37 °C. Cells were treated with increasing concentrations of cisplatin (0–10 μM) for 72 hr, alone or in combination with ATRA (5 μM) or retinol (1 μM), after which time culture media was removed and replaced with fresh treatment-free media and re-incubated for 10 days. Colonies were fixed and stained with 25% (v/v) methanol, 0.05% (w/v) crystal violet for 30 mins. Residual stain was removed by rinsing wells gently with water. Colonies were counted using the ColCount™ colony counter (Oxford Optronix Ltd, Oxford, UK). Plating efficiencies (PE) were calculated using the formula: PE = Number of colonies/Number of cells seeded. The percentage surviving fraction (SF) was calculated using the formula: SF = (PE treated colonies/PE untreated) × 100.

### Apoptosis

Apoptosis was measured by flow cytometry using dual Annexin-V and propidium iodide (PI) staining to identify early and late apoptotic cells from live and necrotic cells. Briefly, cells were seeded in 6-well plates at a density of 1 × 10^5^ cells per well and allowed to adhere overnight. Cells were treated with increasing concentrations of cisplatin (0–100 μM), alone or in combination with ATRA (5 μM) or retinol (1 μM) in cell culture media for 48 hr. Untreated control cells were treated with media only. Following treatment, both floating and adhered cells were collected, transferred to FACS tubes and placed on ice. Cells were pelleted by centrifugation and washed in 1 ml of 1 × Annexin-V binding buffer (BB), pelleted, and re-suspended in 200 µl BB. A volume of 2 µl Annexin-V-FITC (IQ Products) was added to each test sample and incubated at 4 °C for 20 min. After this time, 1 ml of 1 × BB was added to each tube and cells pelleted by centrifugation. Immediately before FACS analysis, cells were re-suspended in 400 µl of 1 × BB containing 1 µg/ml PI (Invitrogen). Apoptotic cells were measured using a CyAn™ ADP flow cytometer (Dako, USA). Forward scatter *versus* side scatter plots were used to eliminate debris, while forward scatter *versus* pulse width plots isolated single cells for analysis.

### Quantitative real-time PCR

Total RNA was isolated from cell lines using TRI Reagent (Molecular Research Center, USA). RNA yield and purity were determined using a Nanodrop-1000 spectrophotometer. Gene expression analysis of CSC markers, retinoic acid receptors (RAR) and retinoid X receptors (RXR) was carried out using the Luna Universal One-Step RT-qPCR Kit (New England BioLabs Inc), in duplicate, on the ABI 7500 qPCR platform. ß-actin was used to normalise for endogenous gene expression. Complementary DNA (cDNA) was generated by reverse transcription for 10 min at 55 °C. Real-time PCR was carried out by denaturation at 95 °C for 1 min followed by 45 cycles consisting of denaturation at 95 °C for 10 s, primer extension at 60 °C for 60 s and a final elongation step at 60 °C for 1 min. Mean Ct values were determined and differences in gene expression levels were calculated as fold-changes relative to controls. Primers were designed using the University of California Santa Cruz (UCSC) Genome Browser and synthesised commercially (Sigma-Aldrich). Primer sequences used are as follows: Nanog *FWD* 5′-TTGGAGCCTAATCAGCGAGGT-3′, *REV* 5′-GCCTCCCAATCCCAAACAATA-3′; Oct-4 *FWD* 5′-ATTCAGCCAAACGACCATCT-3′, *REV* 5′- GTTTTCTTACTAGTCACGTGCGG-3′; Sox-2 *FWD* 5′-GGAGCTTTGCACGAAGTTTG-3′, *REV* 5′- GGAAAGTTGGGATCGAACAA-3′; Klf4 *FWD* 5′-CACACTTGTGATTACGCGGG-3′, *REV* 5′- CCCGTGTGTTTACGGTAGTGC-3′; cMyc *FWD* 5′-CCTCGGATTCTCTGCTCTCCTC-3′, *REV* 5′- AGGTTTGCTGTGGCCTCCAG-3′; CD133 FWD 5′-GAGAAAGTGGCATCGTGCAA-3′, *REV* 5′- CACGTCCTCCGAATCCATTC-3′; RARα *FWD* 5′-AGATCACCCTCCTCAAGGCTG-3′, *REV* 5′-AAGGCAAAGACCAGGTCGGT-3′; RARβ *FWD* 5′-GATCTCCGTAGCATCAGTGC-3′, *REV* 5′-GTGTTCCCACTTGAACTTGG-3′; RXRα *FWD* 5′-CCTTGGAGGCCTACTGCAAGC-3′, *REV* 5′- TGTGTCCCCGATGAGCTTGA-3′.

### Flow cytometry and fluorescence-activated cell sorting (FACS)

H460, H1299 and SKMES-1 PT and CisR NSCLC cells were collected for FACS analysis for human CD44, CD133 and epithelial cell adhesion molecule (EpCAM) surface marker expression and divided into unstained, isotype (IgG1) controls and phycoerythrin (PE)-conjugated test antibodies (Miltenyi Biotec). Briefly, samples were centrifuged at 1300 rpm for 3 min and pellets were washed in 1 ml FACS buffer (PBS, 0.01% sodium azide, 2% FBS), vortexed and centrifuged. Supernatants were discarded and cells were re-suspended in 100 μl FACS buffer and appropriate samples were stained with 10 μl antibody and incubated at 4 °C for 10 min in the dark. Following incubation, FACS buffer (2 ml) was added to the cells and samples were centrifuged for 10 min at 1300 rpm. Cells were re-suspended in 500 µl FACS buffer and stored on ice in the dark. Cells were analysed on the FL2 channel on a CyAN™ ADP flow cytometer (Beckman Coulter, CA, USA) using Summit software (v4.3). Cells were distinguished from debris using a forward scatter *versus* side scatter plot. Single cells were separated from doublet and triplet cell complexes using a forward scatter *versus* pulse width plot. Test antibodies were compared to the negative mouse IgG1 isotype control antibodies to assess and quantify positively stained cell populations.

### Statistical analysis

Analysis between groups was carried out using analysis of variance (ANOVA). Statistical comparison of two means was carried out using an unpaired, two-tailed Student's *t*-test. Significance was defined as *p* ≤ 0.05. Data are graphically represented as Mean ± Standard Error of the Mean (SEM). All data were analysed using GraphPad Prism™ (version 5) statistical software.

## Results

### ALDH1 activity is increased in chemo-resistant NSCLC cells

A panel of isogenic cisplatin-resistant NSCLC cell lines were previously generated and characterised in our laboratory [40]. A cisplatin resistant phenotype was demonstrated in this model of chemoresistance as shown by a significantly decreased proliferative capacity of lung tumour cells in response to cisplatin, a significantly increased resistance to cisplatin-induced cell death, accumulation of resistant cells in the G0/G1 phase of the cell cycle and significantly enhanced clonogenic survival ability of the cisplatin resistant sublines. Furthermore, resistant cells exhibited a putative stem-like signature with increased expression of CD133+/CD44+ cells and significantly increased ALDH1 activity relative to their age-matched corresponding parental cells. While cisplatin resistant sublines demonstrated decreased uptake of cisplatin in response to treatment, reduced cisplatin-GpG DNA adduct formation and a significant decrease in γH2AX foci were observed relative to control/parental cell lines.

**Fig. 1:**
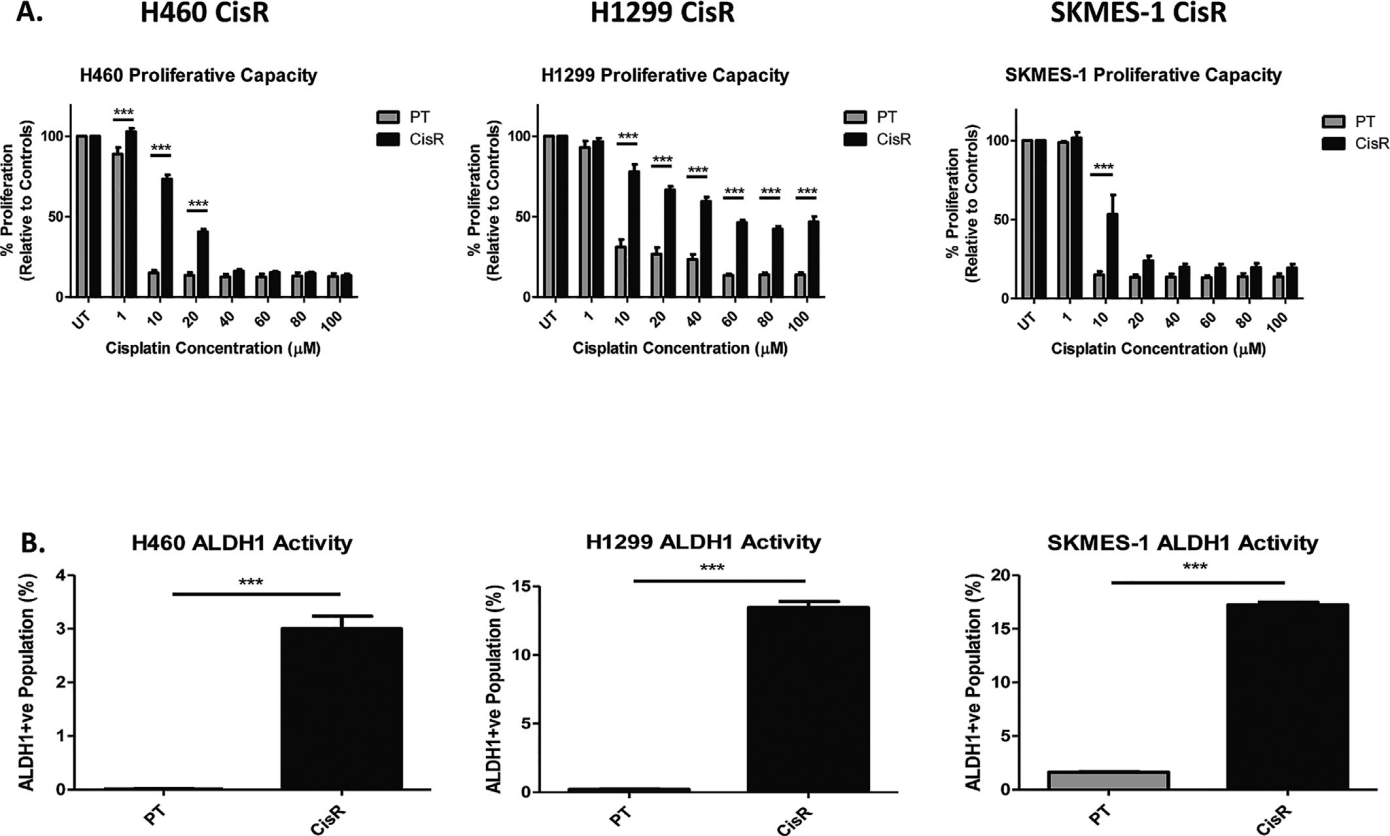
Cisplatin resistant NSCLC cells exhibit increased proliferation and ALDH1 activity.

In dose-response studies (Fig. 1A), both the cisplatin resistant sublines (maintained in culture using their respective IC_50_ cisplatin concentrations) and matched parental cell lines, were treated with increasing concentrations of cisplatin. The cytotoxic effects of cisplatin on cell proliferation showed significant differences between the resistant and corresponding parental cell lines, with significantly more cell kill in the parental (sensitive) cells relative to their cisplatin resistant counterparts. Indeed, differential responses were observed across all three cell lines examined (H460, H1299, SKMES-1), representing the different NSCLC histologies of large cell carcinoma, adenocarcinoma and squamous cell carcinoma, respectively. Relative to H460 and SKMES-1 cell lines, H1299 cells were significantly more resistant to cisplatin. This may be due to a greater inherent resistance in these cells. These observations reflect, at least in part, that observed in the clinical setting where advanced stage NSCLC patients treated with cisplatin-doublet chemotherapy eventually relapse during treatment due to acquired resistance to this platinum drug. These findings are further supported by peak plasma concentrations of cisplatin (3–5μg/ml) following intravenous infusion in lung cancer patients, which are equivalent to 10-15μM cisplatin *in vitro*.

The Aldefluor assay was used to investigate the presence of a subpopulation of cells with ALDH1 activity within the NSCLC panel of PT and CisR cell lines. A significant increase in ALDH1 activity was identified across all CisR sublines examined relative to their PT counterparts. The Aldefluor assay identified a distinct ALDH1+ve subpopulation relative to DEAB-inhibited controls in all cell lines, but with no detection in the H460 PT cells (Fig. 1B). The H460 CisR subline showed a significant and distinct ALDH1+ve subpopulation relative to PT cells and internal DEAB control (3.00 ± 0.23% *vs* 0.00 ± 0.01%, *p*<0.001), similar to that found in H1299 CisR cells (13.46 ± 0.44% *vs* 0.20 ± 0.04%, *p*<0.001) and SKMES-1 CisR cells (17.22 ± 0.25% *vs* 1.63 ± 0.05%, *p*<0.001). Dot plots showing gating of ALDH1+ve and ALDH1-ve subpopulations in H460, H1299 and SKMES-1 CisR cells are shown **(Supplementary Fig. 1).** These data indicate a subpopulation of cisplatin-resistant lung cancer cells that are enriched for an ALDH1+ve cell subset.

### ATRA and retinol deplete ALDH1+ve subpopulations in cisplatin-resistant NSCLC cells

**Fig. 2:**
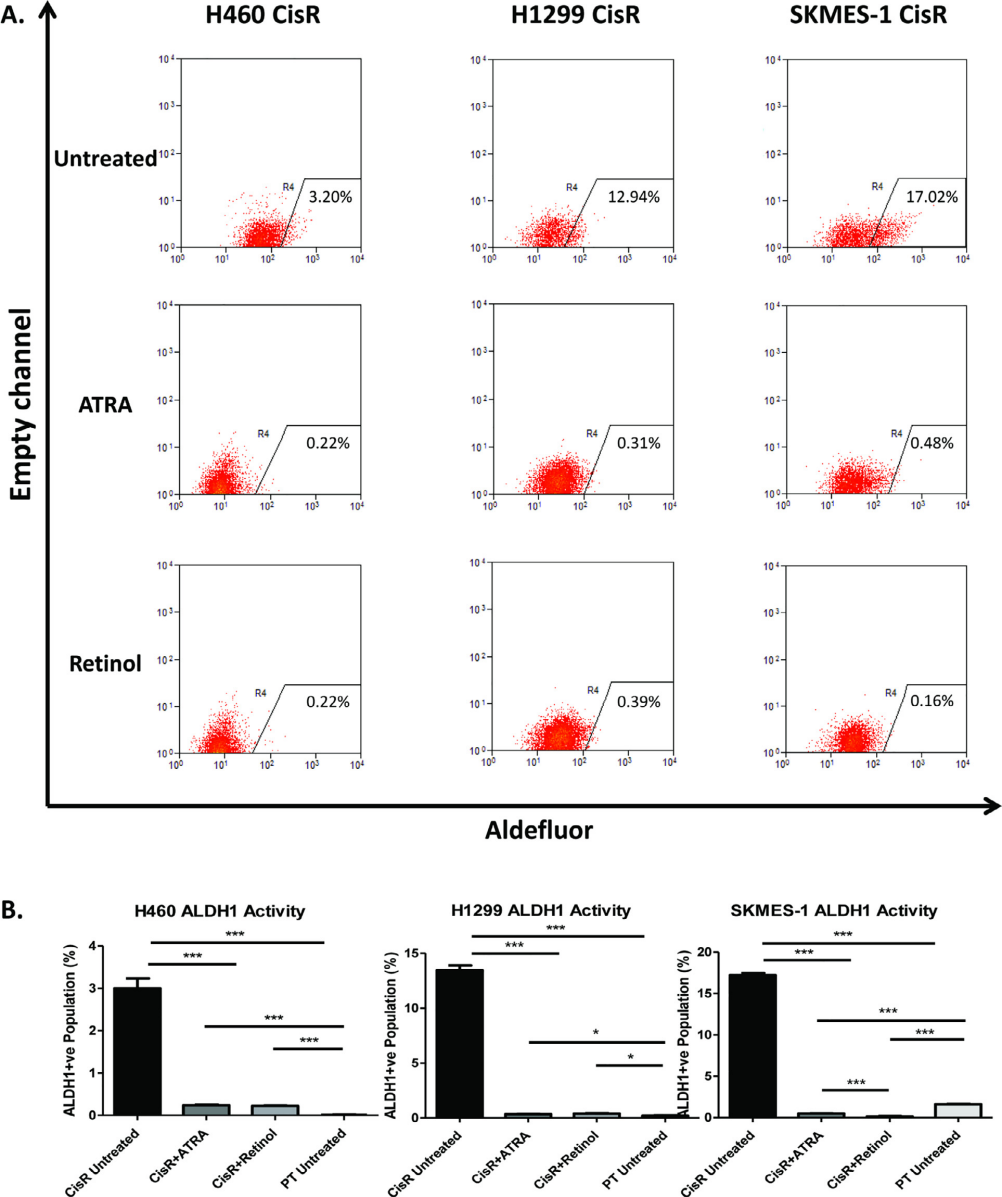
Products and substrates of the retinoic acid pathway inhibit ALDH1-positive CSC populations in cisplatin resistant cells.

Cisplatin-resistant cell lines were treated with ATRA (5 μM), the final product of the retinoic acid pathway, or retinol (1 μM), an early substrate of the retinoic acid pathway, to investigate the potential of exploiting the vitamin A/retinoic acid axis to induce differentiation of characterised ALDH1+ve CSC subpopulations in an *in vitro* model of cisplatin resistance. Relative to untreated cells, cisplatin-resistant cell lines were treated with ATRA or retinol and the presence of an ALDH1+ve subpopulation was assessed by flow cytometry using the aldefluor assay. FACS plots and gating are represented (Fig. 2A). At baseline, both ATRA **(Supplementary Fig. 2)** and retinol **(Supplementary Fig. 3)** significantly reduced ALDH1+ cell populations in cisplatin resistant sublines relative to untreated cisplatin resistant cells. While there was little if no ALDH1+ cell populations detected in H460 or H1299 parental (sensitive) cell lines, ATRA and retinol significantly reduced this ALDH1+ cell population in parental cells relative to untreated controls, albeit at much lower levels.

Treatment of cisplatin-resistant cell lines with components of the retinoic acid pathway (ATRA and retinol) significantly reduced the ALDH1+ve subpopulation across NSCLC cells representing large cell (H460), adenocarcinoma (H1299) and squamous cell (SKMES-1) histology (Fig. 2B). Treatment of the H460 CisR subline with ATRA significantly reduced the presence of the ALDH1+ve subpopulation relative to untreated cells (3.00 ± 0.23% *vs* 0.21 ± 0.01%, *p*<0.001). A similar reduction of this cell subset was observed in H460 CisR cells in response to retinol (3.00 ± 0.23% *vs* 0.23 ± 0.01%, *p*<0.001). Interestingly, no significant difference was observed in the ALDH1+ve subpopulation of H460 CisR cells when treatment with ATRA and retinol were compared.

A similar trend was observed in the highly metastatic H1299 CisR subline, in which both ATRA (13.46 ± 0.44% *vs* 0.34 ± 0.02%, *p*<0.001) and retinol (13.46 ± 0.44% *vs* 0.41 ± 0.03%, *p*<0.001) significantly reduced the ALDH1+ve subpopulation relative to untreated controls. While these were comparable with the H1299 PT cell line, the ALDH1+ve subpopulation remained significantly greater in the CisR subline following treatment with either ATRA or retinol. Similar to H460 CisR cells, there was no significant difference in the percentage of H1299 CisR ALDH1+ve CSCs observed in response to treatment with ATRA or retinol.

Treatment of the SKMES-1 CisR subline with ATRA (*p*<0.001) or retinol (*p*<0.001) significantly decreased the ALDH1+ve subpopulation relative to untreated CisR cells. However, in contrast to H460 and H1299 CisR cell lines, treatment of the SKMES-1 CisR subline with retinol resulted in a significantly greater decrease in the ALDH1+ve CSC population compared to that observed in response to treatment with ATRA (0.15 ± 0.03% *vs* 0.51 ± 0.02%, *p*<0.001). Both ATRA (*p*<0.001) and retinol (*p*<0.001) significantly reduced the CisR ALDH1+ve subpopulation to levels below that observed in the SKMES-1 cisplatin-sensitive (PT) cell line. Taken together, these data suggest that exploitation of the retinol/retinoic acid axis in cisplatin-resistant NSCLC cells may deplete the ALDH1+ve CSC subpopulation to levels comparable with their cisplatin-sensitive counterparts.

### Supplementation of chemo-resistant lung cancer cells with components of the retinoic acid pathway re-sensitises cells to the cytotoxic effects of cisplatin

**Fig. 3:**
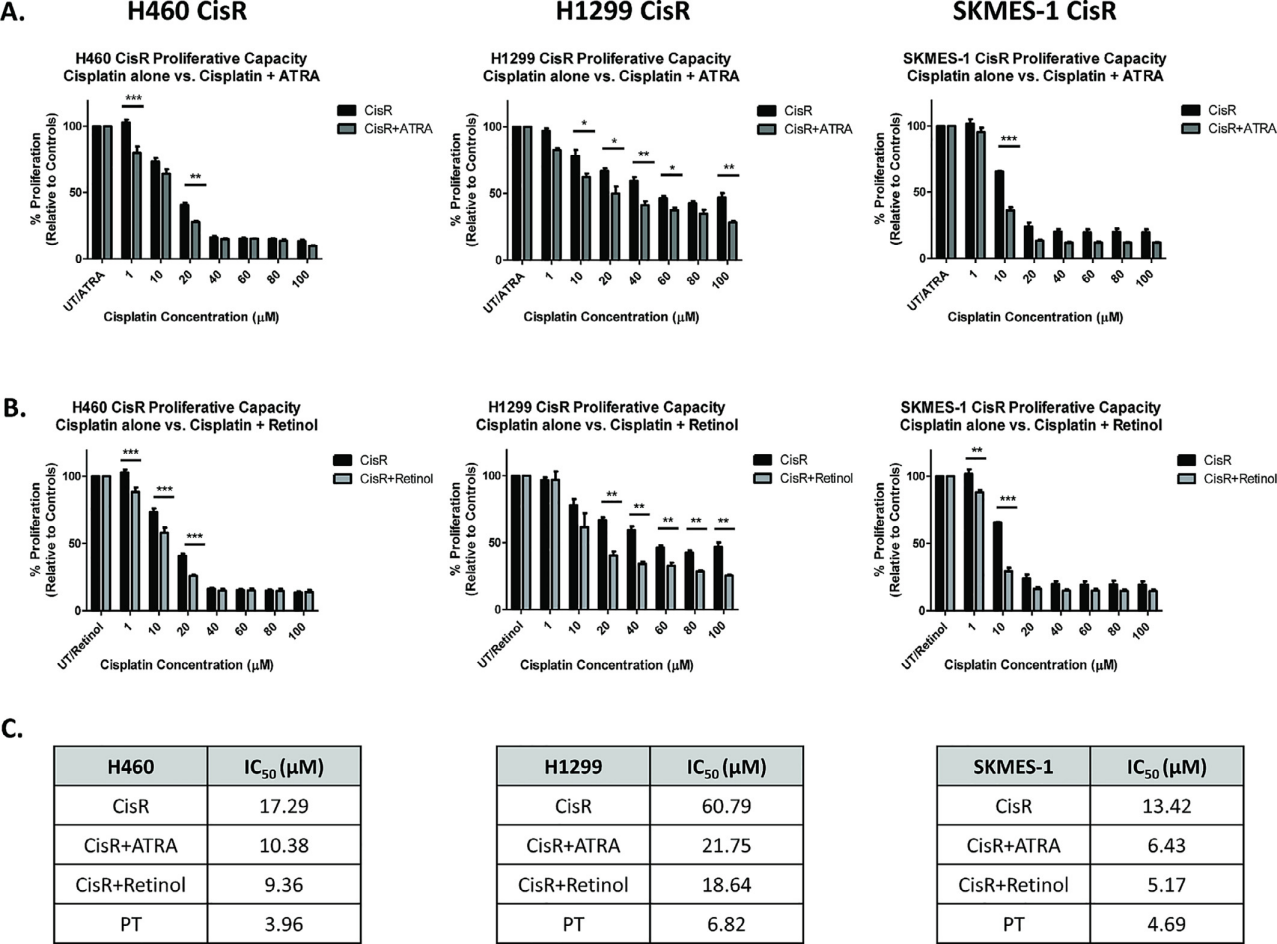
ATRA and retinol enhance the cytotoxic effects of cisplatin.

Based on the above findings, it is now known that treatment of cisplatin-resistant NSCLC sublines with ATRA and retinol significantly inhibits ALDH1 activity and reduces the ALDH1+ve CSC subpopulation. The effects of inhibiting the retinoic acid pathway in cisplatin-resistant lung cancer cells, were further assessed in response to treatment with cisplatin. Cisplatin-resistant sublines were treated with increasing concentrations of cisplatin (0–100 μM) alone or in combination with 5 μM ATRA (Fig. 3A) or 1 μM retinol (Fig. 3B) for 72 hr and their proliferative capacity assessed. Treatment of the H460 CisR subline with ATRA in combination with cisplatin (1 μM and 20 μM) significantly reduced the proliferative capacity of these cells relative to cells treated with cisplatin alone. A similar response was observed in H1299 CisR cells in response to a wide range of cisplatin concentrations (10–100 μM). In SKMES-1 CisR cells, ATRA in combination with cisplatin significantly reduced cell proliferation at 10 μM. Similar effects on proliferation were observed in H460, H1299 and SKMES-1 CisR sublines following treatment with retinol and cisplatin as a combined treatment modality.

Mean IC_50_ concentrations were determined for each cell line using log transformation of the dose-response curves in the presence or absence of treatments (Fig. 3C). Treatment with both ATRA and retinol greatly reduced the IC_50_ concentrations for cisplatin across all resistant sublines. Interestingly, retinol/vitamin A supplementation of cisplatin treatments further reduced these IC_50_ concentrations when compared to ATRA alone. These findings suggest that treatment of cisplatin-resistant NSCLC cells with either ATRA, or retinol, in combination with cisplatin chemotherapy, significantly enhances the anti-proliferative effects of cisplatin. This marked increase in cytotoxicity may occur as a result, at least in part, of the re-sensitising effects of ATRA or retinol on the response of NSCLC cells to cisplatin.

### Clonogenic survival of cisplatin-resistant cells is altered in response to treatment with ATRA and retinol

**Fig. 4:**
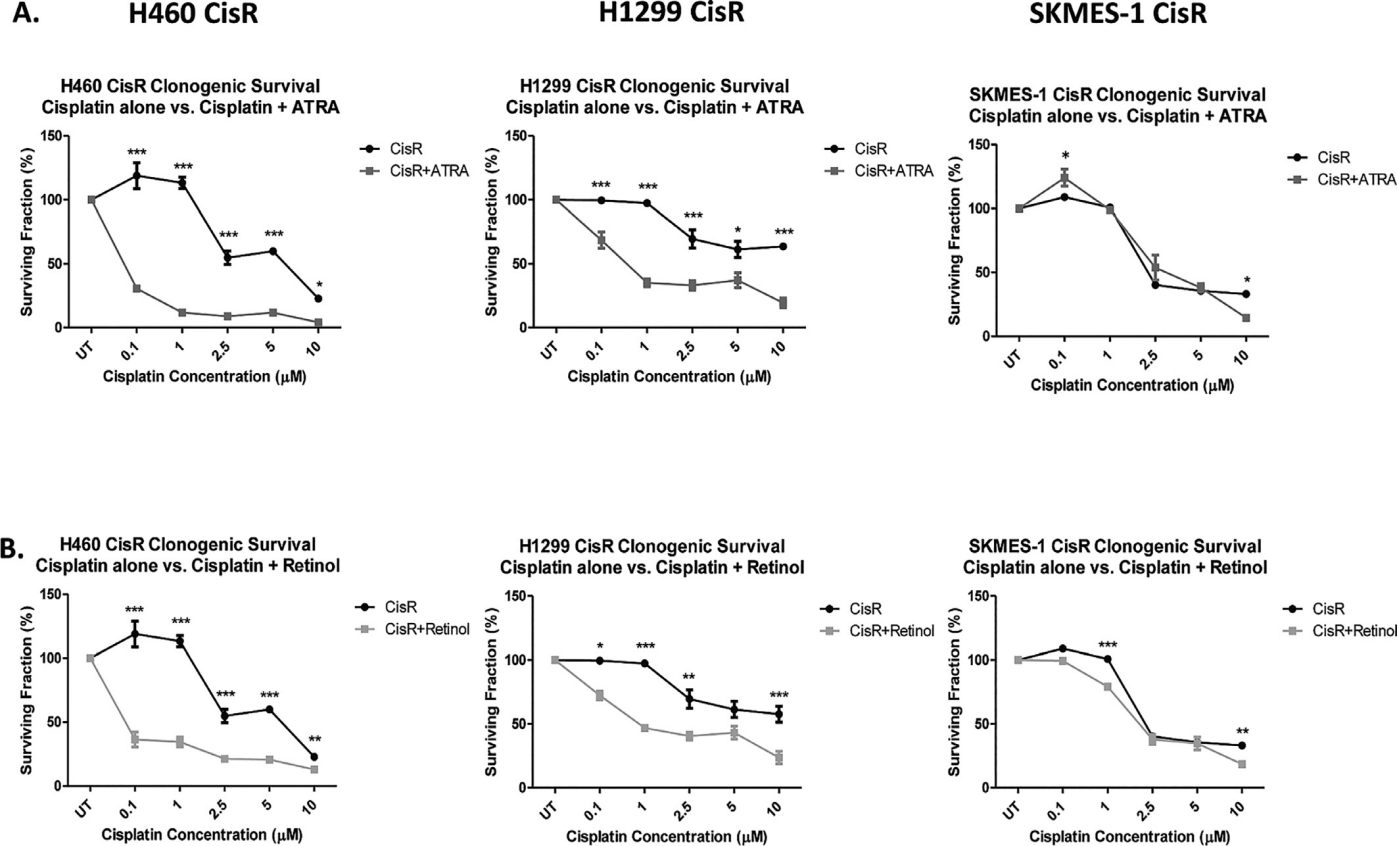
Clonogenic survival is significantly altered in cisplatin resistant lung cancer cells in response to supplementation of vitamin A/retinoic acid pathway components.

Using the clonogenic, or colony formation assay, *in vitro* cell survival ability of H460, H1299 and SKMES-1 CisR cells was assessed following treatment with ATRA (Fig. 4A) or retinol (Fig. 4B) and in combination with increasing concentrations of cisplatin. ATRA, in combination with cisplatin, at all concentrations examined (0.1–10 μM) significantly reduced the clonogenic survival ability of H460 and H1299 CisR sublines compared to cisplatin alone at similar concentrations. In SKMES-1 CisR cells, the ATRA-cisplatin combination treatment significantly decreased the percentage surviving fraction in response to treatment with 10 μM cisplatin relative to cisplatin-only treated cells.

Compared to cisplatin alone, retinol-cisplatin combination treatment significantly decreased the percentage surviving fractions of the H460 and H1299 CisR sublines in response to a range of cisplatin concentrations. Retinol in combination with cisplatin (1 μM and 10 μM) significantly decreased the survival capacity of SKMES-1 CisR cells compared to cisplatin-only controls. Taken together, these data highlight the potential of supplementing current platinum-based therapies with components of the vitamin A/retinoic acid pathway to enhance their cytotoxicity in the context of drug resistance.

### Targeting of the retinoic acid pathway re-sensitises NSCLC cells to cisplatin-induced cell death

**Fig. 5:**
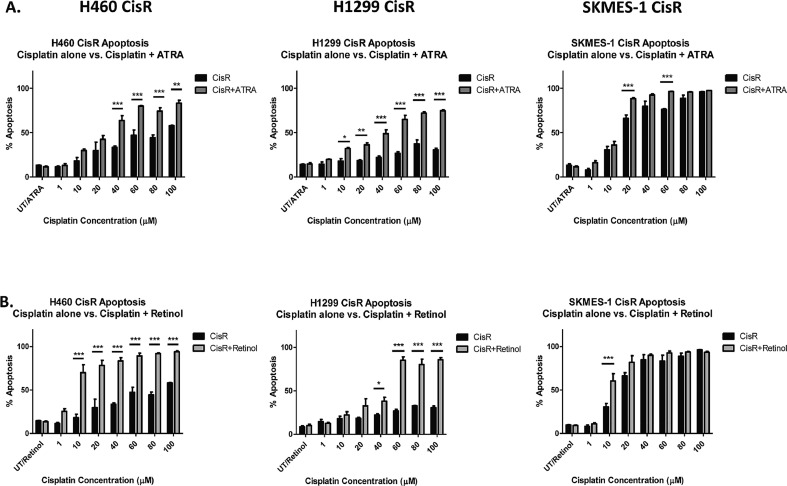
Cisplatin-induced apoptosis of chemo-resistant NSCLC cells is enhanced in response to ATRA and retinol.

To determine the ability of substrates and products of the retinoic acid pathway to re-sensitise resistant cells to cisplatin-induced cell death, apoptosis was measured by flow cytometry following treatment with increasing concentrations of cisplatin (0–100 μM) alone or in the presence of ATRA (Fig. 5A) or retinol (Fig. 5B).

Treatment of cells with ATRA as a single therapeutic agent did not induce apoptosis of the cisplatin-resistant sublines examined. Therefore, any increased cytotoxicity of ATRA-cisplatin combination therapy relative to cisplatin treatment alone, was attributable to the combination treatment of ATRA with cisplatin. Treatment of the H460 CisR cell line with ATRA and increasing concentrations of cisplatin (40–100 μM) significantly enhanced the cytotoxic and pro-apoptotic effects of cisplatin when compared to cisplatin alone at similar concentrations. ATRA potentiated a significant pro-apoptotic effect in H1299 CisR cells in combination with cisplatin (10–100 μM). Similar effects were observed following combination treatment of SKMES-1 CisR cells with ATRA and cisplatin (20 μM and 60 μM).

In examining the response of cisplatin-resistant lung cancer cells to treatment with retinol, this had no effect on apoptosis when used alone as a single agent. Retinol supplementation of the H460 CisR cell line in combination with cisplatin resulted in significantly enhanced sensitivity to cisplatin-induced apoptosis at different cisplatin concentrations (10–100 μM). A similar effect was observed in H1299 CisR cells. Combination treatment of H1299 CisR cells with retinol and cisplatin (40–100 μM) significantly increased apoptosis. The percentage of apoptotic H1299 CisR cells when treated with 100 μM of cisplatin alone was 30.62 ± 1.9%, whereas when combined with retinol, apoptotic cell death of resistant cells significantly increased to 85.78±2.49%. Retinol supplementation of the SKMES-1 CisR subline increased cisplatin-mediated cell death when used in combination with 10 μM cisplatin compared to cisplatin alone at the same concentration. Representative dot plots showing early (R6) and late (R4) stage apoptosis in response to ATRA (5 μM) and different concentrations of cisplatin across H460, H1299 and SKMES-1 cisplatin resistant NSCLC sublines are shown **(Supplementary Fig. 4).**

### ATRA induces differential gene expression of the retinoic acid (RAR) and retinoid X (RXR) nuclear receptors in cisplatin-resistant lung cancer cells

**Fig. 6:**
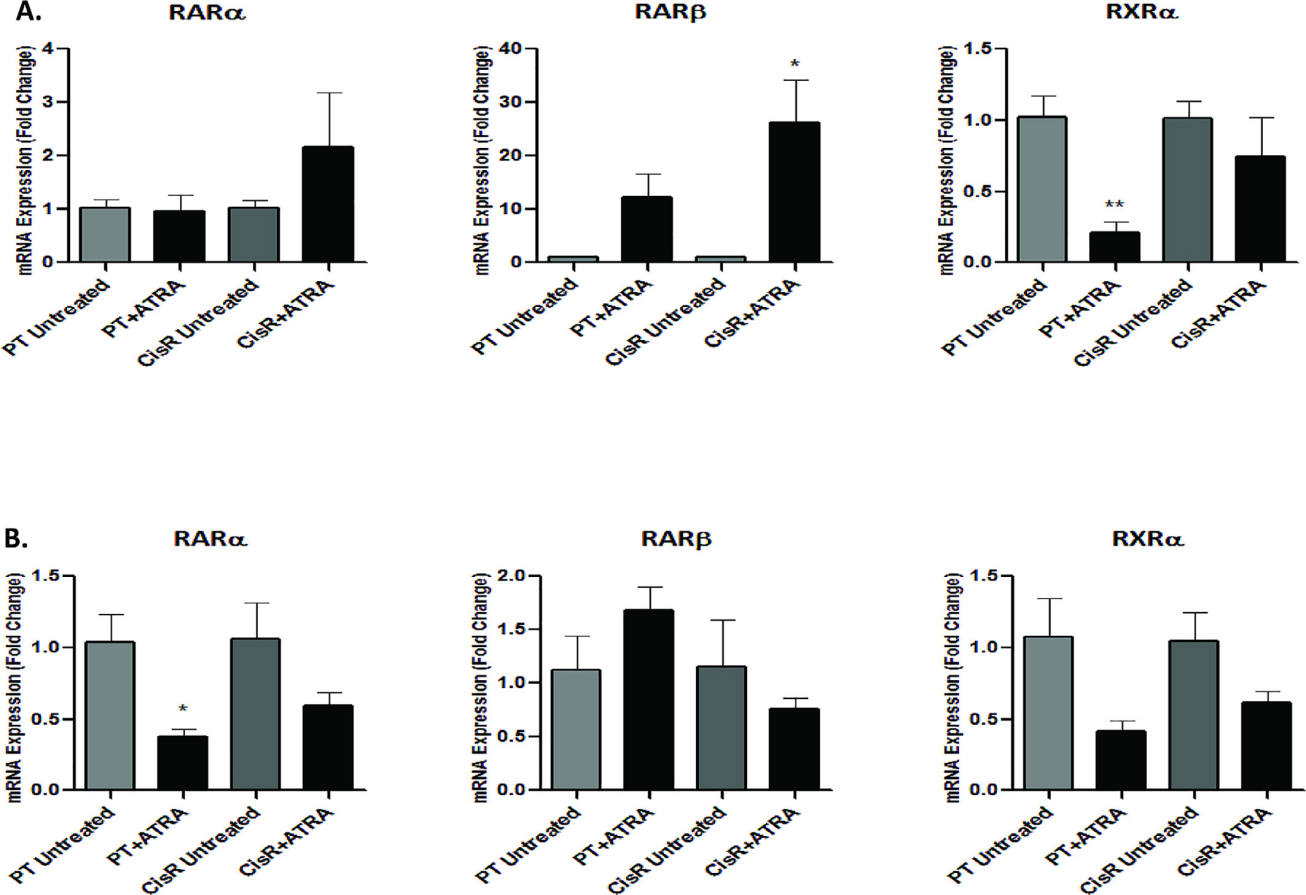
Expression of the retinoic acid and retinoid X receptors in NSCLC cells is altered in response to ATRA.

ATRA is an active metabolite that regulates the expression of target genes through the binding and activation of different nuclear RAR and RXR receptor isoforms, thereby inhibiting proliferation and inducing differentiation of malignant cells. Treatment of H1299 and SKMES-1 PT and CisR sublines with ATRA induced differential expression of RARα, RARβ and RXRα mRNA (Fig. 6). Of interest, while ATRA significantly decreased RARα and RXRα expression in SKMES-1 (*p* = 0.03) and H1299 (*p* = 0.0079) PT cells, respectively, there was a significant increase in the tumour suppressor and major target gene of retinoid action, RARβ, in H1299 CisR cells (*p* = 0.0326) relative to untreated controls. Unlike RARα and RXRα receptors, there was little or no baseline expression of RARβ in H1299 PT or CisR cells. Upon treatment with ATRA, expression of this key retinoic acid receptor isoform was significantly induced in the CisR subline. This is an important finding and may account for the significant increase in ATRA-mediated apoptosis observed in the H1299 cisplatin resistant subline when treated in combination with cisplatin. While ATRA induced an increase in RARβ in H1299 PT cells, this was not statistically significant. Further exploratory analysis is warranted at the protein level to confirm these observed changes in RAR and RXR receptors at the gene level.

### Cancer stem cell surface marker expression is altered in cisplatin resistant NSCLC cells

Based on the reported expression of CD44, CD133 and EpCAM as cancer stem cell surface markers, H460, H1299 and SKMES-1 cells were screened to examine differences, if any, in their cell surface expression of these markers between matched PT and CisR cell lines **(Supplementary Fig. 5).** While there was significant increase in CD44 across all PT and CisR cell lines relative to their isotype controls, CD44+ populations were significantly higher in PT cells relative to their CisR counterparts. On the contrary, there were no significant differences in surface marker expression of CD133 across any of the cell lines examined. While there was a trend towards an increase in EpCAM+ cells in H1299 PT and CisR cell lines, a significant increase in EpCAM+ cells was observed in H460 and SKMES-1 PT and CisR cells relative to isotype controls. Of interest, was the finding that CisR cells demonstrated a significantly lower EpCAM+ cell population relative to their corresponding PT cells. This heterogenous expression of EpCAM in NSCLC cells may be related to the EMT process whereby epithelial tumour cells often undergo epithelial-mesenchymal transition (EMT). During this process, cells undergo phenotypic changes such as loss of epithelial marker expression and as a result, acquire a stem cell-like phenotype.

### ALDH1-positive cell fractions display increased stemness marker expression

**Fig. 7:**
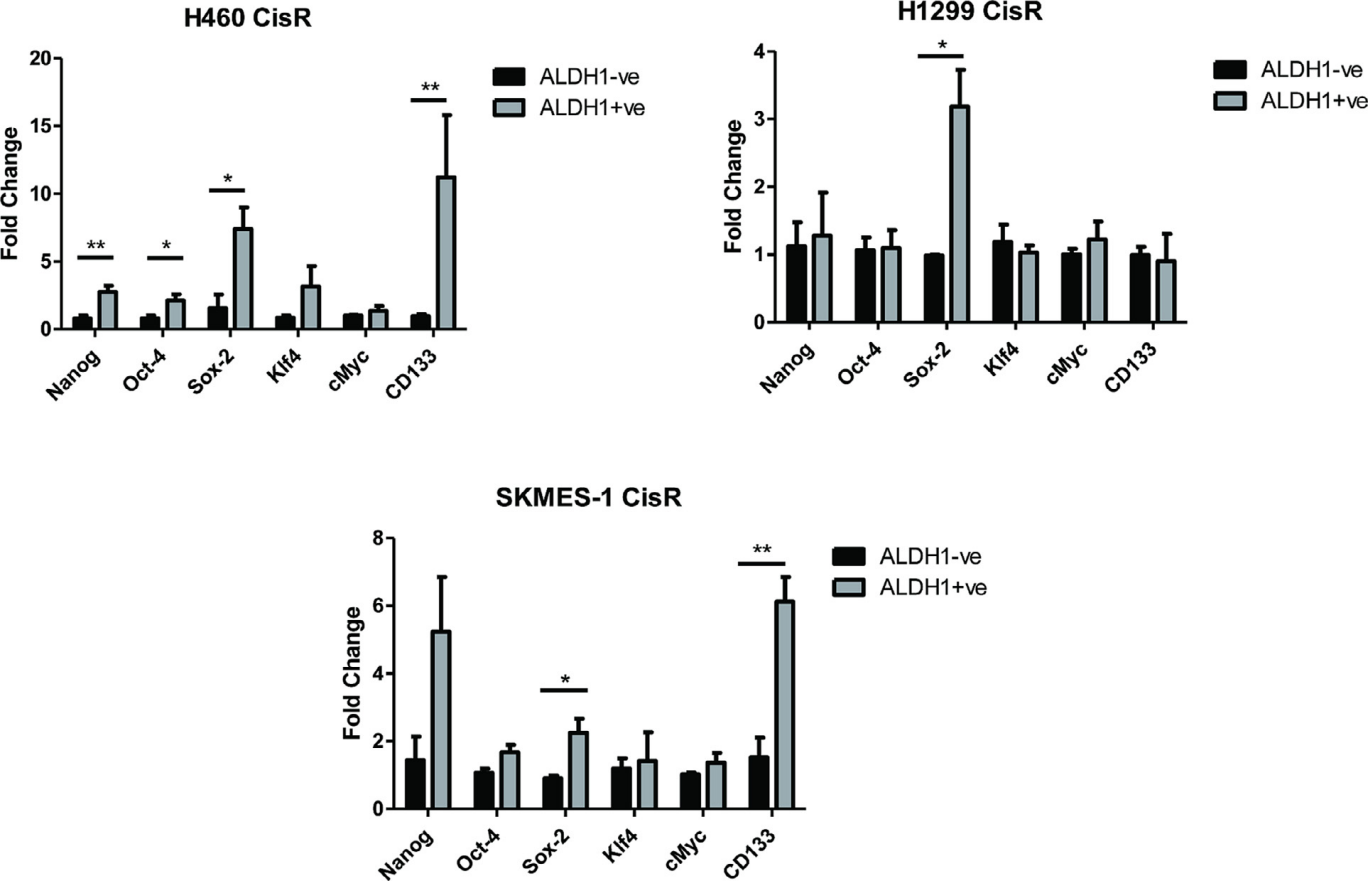
Gene expression profile of stemness markers is altered in cisplatin resistant ALDH1-positive cell fractions.

mRNA expression of the embryonic stem cell factors (Nanog, Oct-4, Sox-2, Klf4 and cMyc) and CSC marker, CD133, were examined at the gene level to elucidate the stemness phenotype of ALDH1+ve subpopulations derived from H460, H1299 and SKMES-1 CisR sublines relative to their ALDH1-ve counterparts (Fig. 7). In H460 ALDH1+ve cells, Nanog (*p* = 0.0052), Oct-4 (*p* = 0.0364), Sox-2 (*p* = 0.0179) and CD133 (*p* = 0.0036) genes were significantly overexpressed. While there was a trend towards increased expression of Klf4 and cMyc mRNA in this ALDH1+ve cell population, this did not reach statistical significance (*p* = 0.5081 and 0.4378, respectively). Of the various stemness genes examined in ALDH1+ve and ALDH1-ve cell fractions from H1299 CisR sublines, Sox-2 (*p* = 0.0265) was the only marker significantly altered between both ALDH1 cell fractions. A similar increase in Sox-2 (*p* = 0.0178), in addition to the CSC marker CD133 (*p* = 0.004), was observed in SKMES-1 ALDH1+ve cells. To confirm changes in protein expression for these cancer stemness genes, protein analysis is warranted as these may be regulated by post-translational modifications.

## Discussion

Cancer stem cells have been described in many solid tumour types and have been characterised by their potential to survive chemotherapy treatment, initiate and maintain tumour growth, give rise to heterogeneous lineages via self-renewal and differentiation in addition to their high expression of stem cell-associated genes [41,42]. These unusual features identify them as prime suspects in tumour relapse, metastasis and subsequent resistance following initial chemotherapy exposure. We have identified the presence of a robustly resistant ALDH1+ve subpopulation of cells within our isogenic model of cisplatin-resistant NSCLC cell lines (H460, H1299 and SKMES-1). Previous characterisation of these ALDH1+ve subpopulations identified a number of key properties typical of cancer stem cells, such as increased gene expression of the pluripotent stem cell markers, Nanog, Oct-4, Sox-2, Klf4, cMyc and CD133, the ability to asymmetrically divide and give rise to differentiated lineages, expansion of an ALDH1+ve subpopulation during platinum exposure and increased resistance to cisplatin [27].

Targeted inhibition of CSCs in combination with conventional chemotherapeutic agents holds great promise as a strategy to overcome chemo-resistance, tumour relapse and metastasis. However, specific targeting of CSCs has not been extensively explored as a therapeutic strategy in the treatment of lung cancer. Specific inhibition of CSC-associated drug transporter pumps such as ABCG2 using Axitinib, has been shown to enhance the efficacy of chemotherapeutic agents in CSC cells [43]. Axitinib is a potent oral small molecule, ATP competitive, multi-target tyrosine kinase inhibitor that inhibits the CSC marker CD117 and ABCG2. Axitinib has been shown to reverse multidrug resistance via ABCG2 inhibition *in vitro* and *in vivo*. Axitinib-doxorubicin combination treatment promoted intracellular accumulation of doxorubicin within CSC side populations and significantly enhanced the cytotoxic effects of doxorubicin [43].

More recently, there has been a significant focus on the inhibition of ALDH1 in the targeting of CSCs to overcome drug resistance [44]. Conventional chemotherapy has demonstrated little effect in the treatment of malignant pleural mesothelioma. However, combination treatment of ALDH^high^/CD44^+^ subpopulations isolated from three mesothelioma cell lines (H28, H2052 and Meso4) using the ALDH1 inhibitor, diethylaminobenzaldehyde (DEAB), induced cisplatin sensitivity in these cells and significantly reduced cell viability [45]. siRNA-mediated knockdown or chemical inhibition of ALDH1A1 in A549 and H522 lung cancer cell lines decreased their proliferative and migratory capacities [46]. More recently, it has been shown that the repurposing of the FDA-approved ALDH1 inhibitor Disulfiram (Antabuse), originally used in the treatment of chronic alcoholism, has potential in the treatment of breast, cervical, prostate, melanoma and lung tumours [47], [48], [49]. Studies have shown that Disulfiram re-sensitises tumour cells to current therapies and enhances the cytotoxic effect of anti-cancer treatments such as platinum-based agents and radiation therapy [27,^50^,51]. The potential use of Disulfiram as a pan-ALDH1 inhibitor was highlighted in a recent Phase IIb trial, in which Disulfiram in combination with cisplatin and vinorelbine was examined in the treatment of metastatic NSCLC patients. The drug was well tolerated and significantly prolonged overall patient survival [52]. Interestingly, of the forty patients included in the trial, there were two long-term survivors, both of whom were in the Disulfiram-chemotherapy combination treatment group.

Our previous identification of an ALDH1+ve CSC subpopulation in cisplatin-resistant NSCLC cell lines lead us to investigate the association of ALDH1 with the retinoic acid pathway [27]. ALDH1 is an enzyme that plays a key role in the metabolism of vitamin A to its metabolite, retinoic acid, a crucial modulator of cell differentiation [28]. Based on its ability to induce cell differentiation, retinoic acid has long been used as a therapeutic agent in the treatment of APL in its all-*trans* form (ATRA), where it induces the maturation of immature cells to their terminally differentiated state [30]. Combining ATRA with cytotoxic chemotherapy is an attractive strategy where ATRA has demonstrated synergism with chemotherapy *in vitro* facilitating apoptosis in ovarian and head and neck carcinoma cells [53]**.** In a randomised Phase II trial investigating the addition of ATRA to first-line treatment with cisplatin and paclitaxel in advanced NSCLC, ATRA increased response rates and prolonged progression-free survival with an acceptable toxicity profile [54]. Studies have also reported the potential therapeutic use of ATRA in NSCLC, both *in vitro* and *in vivo* [55], [56], [57], in addition to the targeting of cisplatin-induced enrichment of CD133+ tumour initiating cells within the cancer stem cell compartment [38]. Clinical trials investigating the use of ATRA in combination with vaccines and chemotherapy (NCT00601796) or as a chemoprevention agent in combination with Vitamin E (NCT00002586) have been completed in lung cancer.

Based on the findings reported in the current study, the functional effect of ATRA on ALDH1+ve CSCs was investigated using an *in vitro* model of cisplatin-resistant NSCLC. Treatment of the cisplatin-resistant NSCLC sublines with low-dose ATRA (5 μM) significantly depleted the presence of the ALDH1+ve subpopulation across all resistant sublines. This finding was in agreement with the observed feedback inhibition of ALDH1 following increased retinoic acid expression reported by Elizondo et al. [58]. Similarly, ATRA has been shown to prevent cisplatin-induced enrichment of CD133+ tumour initiating cells [38]. Pre-treatment with retinoic acid neutralised cisplatin resistance and reduced the fraction of tumour initiating cells and tumour metastasis. Our findings show that when treated with a combination of ATRA and cisplatin, the cisplatin-resistant sublines displayed significantly decreased proliferative capacity, reduced clonogenic survival and increased apoptosis relative to cells treated with cisplatin alone. These findings substantiate a possible synergistic effect of ATRA when used in combination with conventional cytotoxic chemotherapies, similar to those reported in hepatocellular and breast cancer cell lines [59,60] and extends its differentiation potential to an ALDH1+ve CSC population present across cisplatin-resistant NSCLC subtypes. Emerging pre-clinical data in a Phase II pilot study examining the use of ATRA in combination with paclitaxel in patients with recurrent or metastatic breast cancer [61], reported this combination approach to be well-tolerated. While similar progression and survival was reported for paclitaxel alone, paclitaxel in combination with ATRA showed relatively high rates of stable disease.

An interesting observation during this study was the altered surface expression of the cancer stem cell markers, CD44, CD133 and EpCAM between parent and cisplatin resistant NSCLC cell lines, in addition to stemness markers in ALDH1 cell fractions derived from cisplatin resistant sublines. Cancer cells with an EMT phenotype display reduced EpCAM expression. Furthermore, EMT has been reported to result in cells with stem cell-like properties that display a more drug resistant phenotype, particularly in response to chemotherapeutic agents [62]**.** When compared to H1299 and SKMES-1 PT and CisR cell lines, which demonstrated little or no decrease in EpCAM and minimal changes in stemness gene expression, H460 CisR cells had significantly reduced cell surface expression of EpCAM and a corresponding increase in the stemness marker profile of ALDH1+ve cell fractions relative to ALDH1-ve fractions isolated from the H460 cisplatin resistant subline.

Increased expression of embryonic stem cell factors are now widely associated with resistance to chemotherapeutic drugs used in the treatment of different cancer types in the clinical setting [63], [64], [65]**.** Supporting these observations, at least in part, are recent studies by Modari et al. [66] suggesting the anti-cancer effects of retinoic acid signalling in colorectal cancer via decreased growth of ALDH+ve colon cancer stem cells and increased differentiation of stem cells. One could also speculate that changes in expression of different ALDH isoforms by different NSCLC cell lines and between parental and cisplatin resistant cells may influence the ALDH+ve stem cell population size. Different ALDH1 isoforms have different substrate specificities for retinoic acid ligands and receptors that could induce changes in retinoic acid metabolism, which in turn could potentially affect ALDH1 levels within these cells.

All-*trans* retinoic acid (ATRA) and 9-*cis* retinoic acid (9*cis*RA) are important products of Vitamin A metabolism and play a significant role in the expression of various genes, including the Retinoic Acid Receptor (RAR) and Retinoid X Receptor (RXR), both of which are comprised of α, β and γ isoforms. RAR and RXR heterodimers bind to specific DNA sequences known as the RA response element, in the promoter regions of target genes to modify their expression and play an important role in the regulation of the cell cycle [67]. The expression of RARβ and RXRβ has been reported to be downregulated in NSCLC enabling their ability to evade apoptosis and have been associated with tumour development and prognosis [68]. More importantly, RARβ is a tumour suppressor and a key target gene of retinoid action [69]. Pre-clinical studies have demonstrated the inhibition of cell proliferation, induction of apoptosis and differentiation in NSCLC cells [70].

These findings support a role for RARβ in H1299 and SKMES-1 CisR cells in our study following treatment with ATRA. RARβ expression was significantly induced in H1299 CisR cells in response to ATRA with little or no significant effect in the squamous carcinoma cell line, SKMES-1. This induction was paralleled with significant alterations in the functional effects of ATRA in H1299 CisR cells relative to those observed in SKMES-1 resistant cells via ATRA-mediated induction of apoptosis. Significantly greater tumour cell death of H1299 CisR cells was observed in response to ATRA when used in combination with increasing concentrations of cisplatin (10–100 μM) relative to their parental counterparts. In contrast to SKMES-1 lung cancer cells, the effects of ATRA in re-sensitising CisR cells to cisplatin-mediated apoptosis were markedly less. This may be explained by the induction of the tumour suppressor effects of ATRA-mediated expression of RARβ in H1299 CisR cells. In parallel with this increase in RARβ, ATRA decreased expression of its heterodimer, RXRα. While there was a similar trend towards a decrease in RXRα in H1299 and SKMES-1 CisR cell lines, this was significantly altered in H1299 PT cells. The upregulation of RARβ which correlated with ATRA-induced growth inhibition of NSCLC cells is in agreement with that reported by Li et al. [71] where ATRA significantly inhibited the growth of Calu-6 and H460 cells. While this was accompanied by induction of RARβ expression, it had little effect on the growth of H292, SKMES-1 and H661 lung cancer cell lines in which RARβ expression was not induced. In a recent study by Zhang et al. [72]**,** human mesenchymal stem cells were used as a model for stem cell differentiation and together with a number of cancer cell lines, the cellular consequences of modulating RXRα during cell differentiation was determined, in addition to potential connections with the carcinogenesis process. Overexpression of RXRα in cancer cells inhibited cell proliferation, invasion, and angiogenesis. The authors reported several lines of evidence suggesting that RXRα is fine-tuned during stem cell differentiation. Controlling the level of RXRα or suppressing its activity may be a key requirement during carcinogenesis. Whether this could help drive the transition from a stem cell into a CSC is an interesting hypothesis warranting further investigation.

While ATRA has been widely investigated as a potential anti-cancer therapy, little is known at present regarding vitamin A/retinol, the progenitor substrate of retinoic acid. In this study, we hypothesised that retinol supplementation may have similar effects to ATRA in cisplatin-resistant NSCLC based on the increased expression of ALDH1 in these chemo-resistant sublines, where ALDH1 is a known metabolic component of the conversion of retinol to retinoic acid [27]. Our data indicate a role for retinol in re-sensitising resistant cells to the cytotoxic effects of cisplatin via depletion of ALDH1+ve CSCs. While further studies are warranted to explore the mechanisms of action of retinol in the treatment of drug-resistant lung cancer, to our knowledge, this is the first study highlighting a potential role for retinol supplementation in reversing platinum drug resistance in NSCLC. Our data show that treatment of cisplatin-resistant NSCLC sublines with ATRA and retinol significantly inhibits ALDH1 activity and reduces the ALDH1+ve CSC subpopulation. However, while not examined in the current study, further investigations are warranted to delineate additional mechanistic insights of ATRA or retinol in cancer stem cell-specific (ALDH1+ve) cell subsets. In addition, exploring these effects on cancer stemness markers and their implications on clonogenic survival, apoptosis and RAR/RXR receptor expression in this cisplatin resistant phenotype would add further knowledge to this field of research. Studies are also warranted to examine the timing and sequence of treatments in this *in vitro* model of cisplatin resistant NSCLC, such as the pre-treatment of cisplatin resistant cells with ATRA or retinol prior to treatment with cisplatin.

This study set out to investigate the potential exploitation of the CSC marker, ALDH1, and its role in the metabolism of vitamin A and subsequent conversion to retinoic acid in cisplatin-resistant NSCLC, and to further explore the differentiation potential of retinoic acid in relation to CSCs and CSC-mediated mechanisms of resistance. Taken together, our data provide new knowledge in the field of cisplatin resistant NSCLC and show that targeting vitamin A/retinoic acid signalling in cisplatin resistant lung cancer depletes an ALDH1+ve CSC population, particularly in cells with increased expression of stemness markers and reduced EpCAM expression that may indeed be reflective of EMT, a key process implicated in drug resistance. Further studies to examine specific EMT markers and their response to ATRA/retinol and delineating the function of different ALDH1 isoforms and their binding to RXR and RAR receptors in NSCLC cells are warranted. These findings however, highlight the potential of breaking the link between ALDH1 and retinoic acid signalling as a promising strategy in the treatment of cisplatin resistant NSCLC.

## Author contribution statement

**Lauren MacDonagh:** conceptualisation, investigation, acquisition & analysis of data, drafting of manuscript. **Rhyla Mae Santiago:** investigation & review. **Steven G. Gray:** conceptualisation & review. **Eamon Breen:** flow cytometry & review. **Sinead Cuffe:** manuscript review. **Stephen P. Finn:** manuscript review. **Kenneth J. O'Byrne:** supervision, funding acquisition & review. **Martin P. Barr:** supervision, funding acquisition, drafting and editing of manuscript.

## Declaration of Competing Interest

The authors have no conflicts of interests to declare.

## Appendix Supplementary materials

Image, image 1

Image, image 2

Image, image 3

Image, image 4

Image, image 5
