# Supplementary figures and images for: Exploitation of the vitamin A/retinoic acid axis depletes ALDH1-positive cancer stem cells and re-sensitises resistant non-small cell lung cancer cells to cisplatin
Source: Transl Oncol. 2021 Feb 5;14(4):101025. doi: 10.1016/j.tranon.2021.101025 (PMC7868629; doi:10.1016/j.tranon.2021.101025)

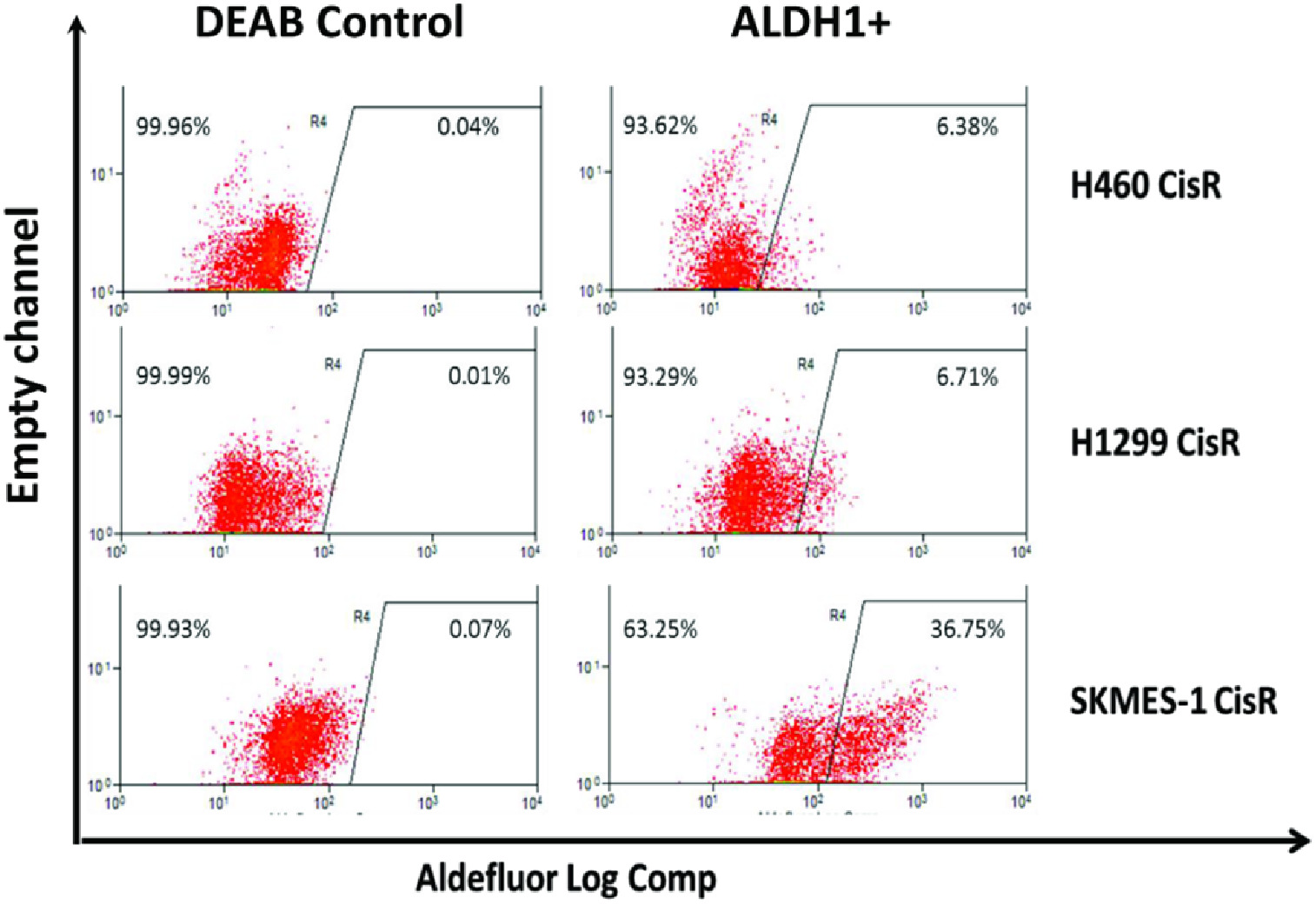

Supplement: Supplementary file 1 [file mmc1.jpg]

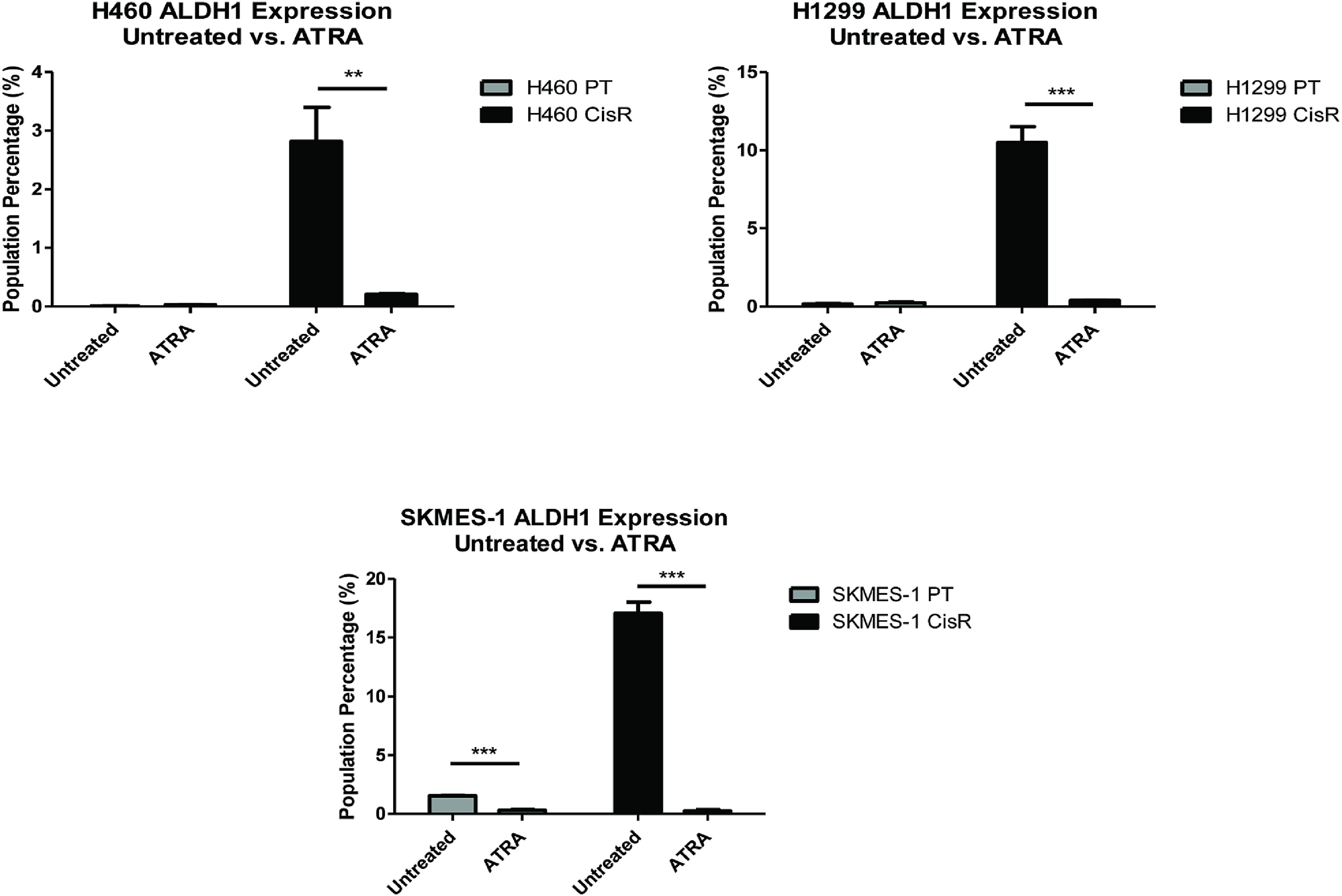

Supplement: Supplementary file 2 [file mmc2.jpg]

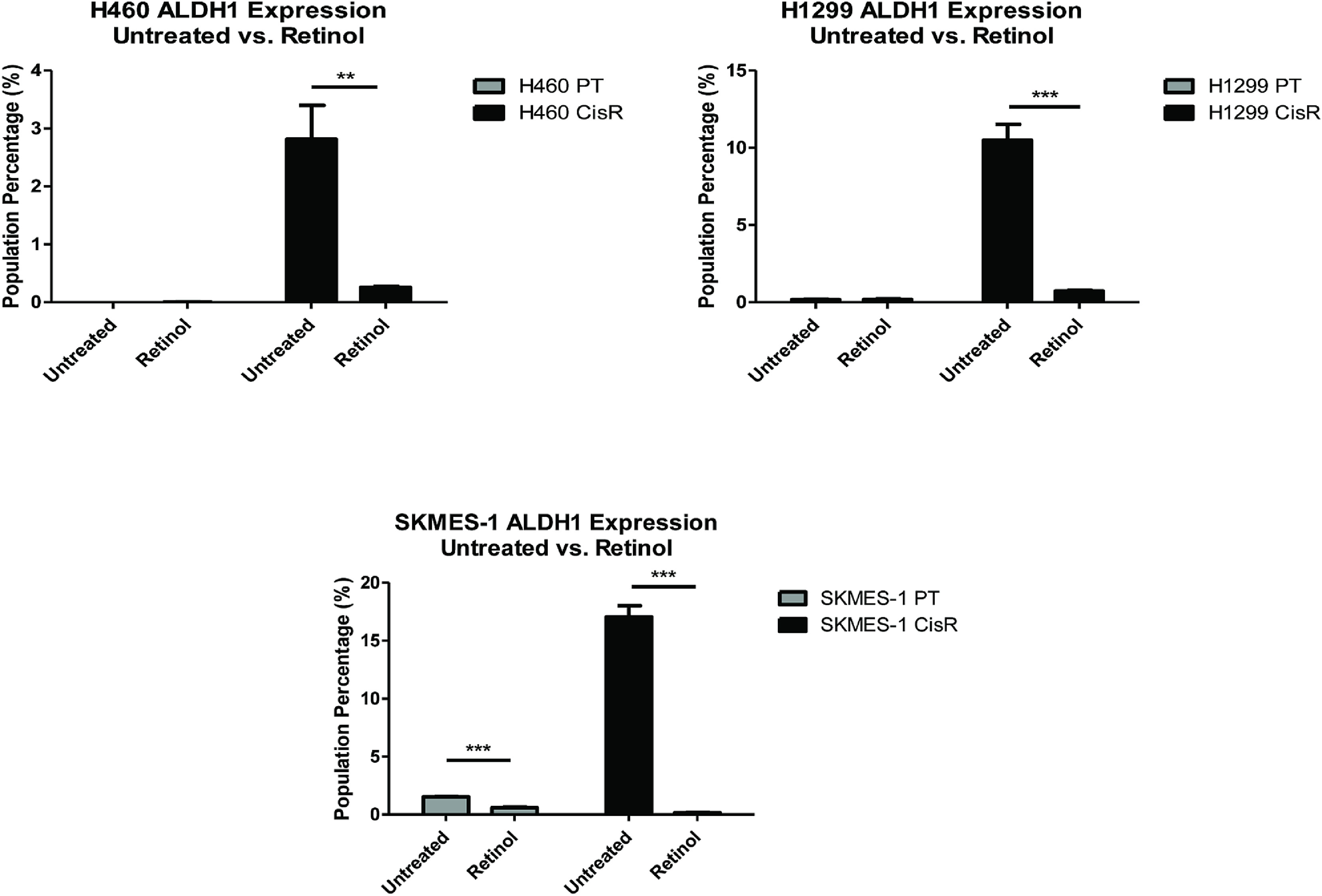

Supplement: Supplementary file 3 [file mmc3.jpg]

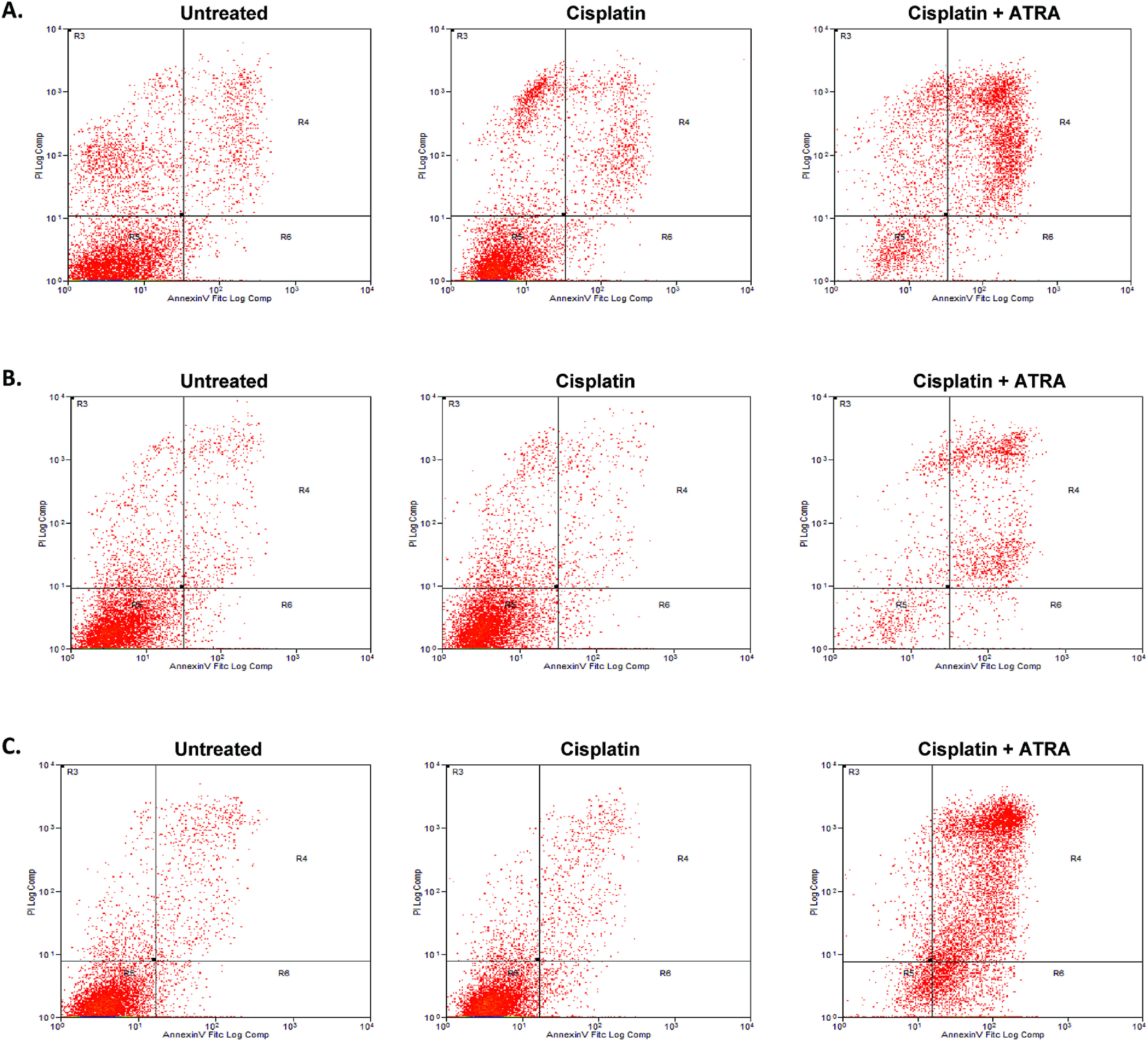

Supplement: Supplementary file 4 [file mmc4.jpg]

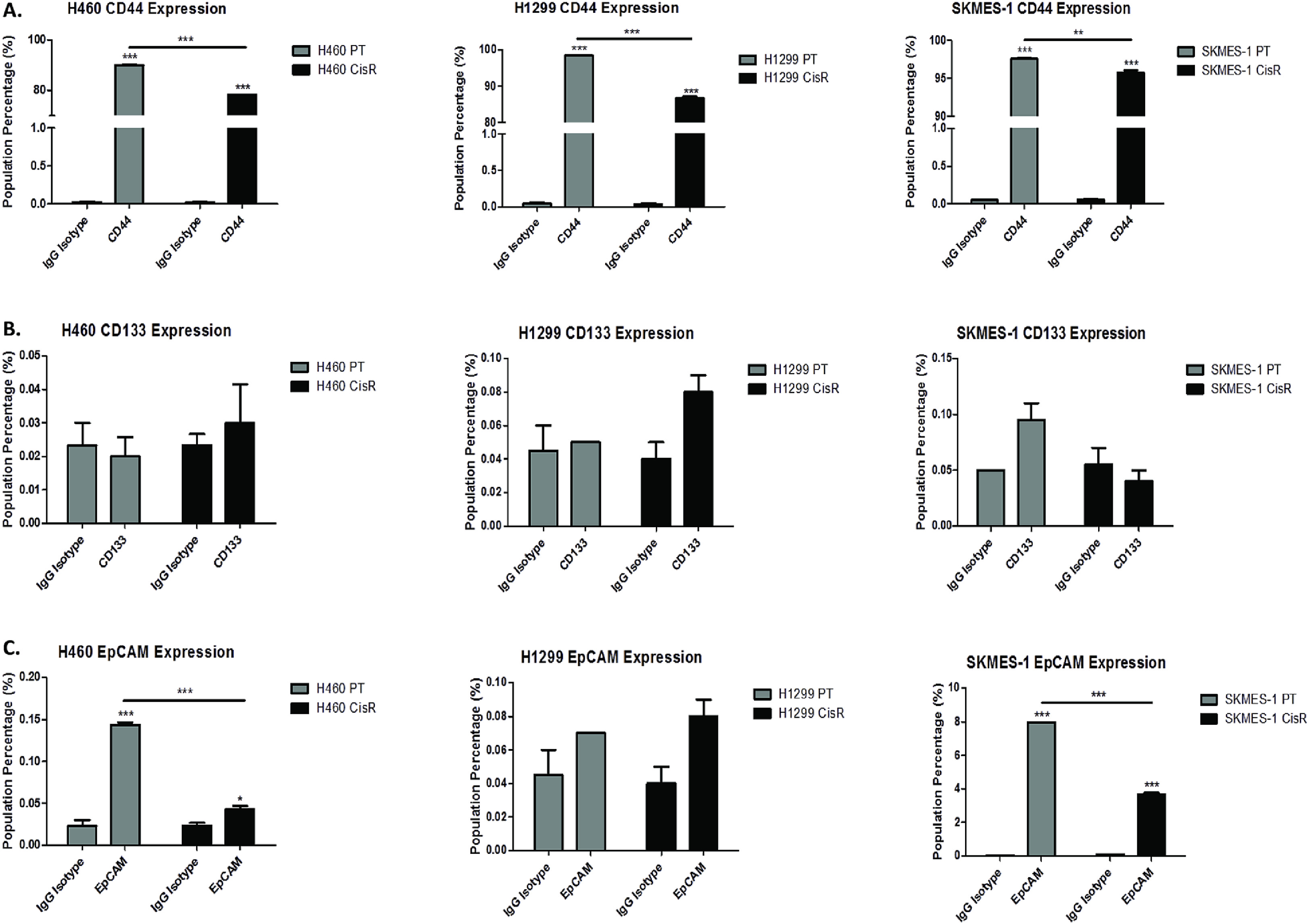

Supplement: Supplementary file 5 [file mmc5.jpg]
